# Using Intermittent Fasting as a Non-pharmacological Strategy to Alleviate Obesity-Induced Hypothalamic Molecular Pathway Disruption

**DOI:** 10.3389/fnut.2022.858320

**Published:** 2022-03-30

**Authors:** Luciana da Costa Oliveira, Gustavo Paroschi Morais, Eduardo R. Ropelle, Leandro P. de Moura, Dennys E. Cintra, José R. Pauli, Ellen C. de Freitas, Rodrigo Rorato, Adelino Sanchez R. da Silva

**Affiliations:** ^1^Postgraduate Program in Rehabilitation and Functional Performance, Ribeirão Preto Medical School, University of São Paulo, São Paulo, Brazil; ^2^Laboratory of Molecular Biology of Exercise, School of Applied Sciences, University of Campinas, São Paulo, Brazil; ^3^School of Physical Education and Sport of Ribeirão Preto, University of São Paulo, São Paulo, Brazil; ^4^Postgraduate Program in Molecular Biology, Laboratory of Stress Neuroendocrinology, Department of Biophysics, Paulista Medical School, Federal University of São Paulo, São Paulo, Brazil

**Keywords:** intermittent fasting (IF), hypothalamus, obesity, hypothalamic inflammation, non-pharmaceutical intervention

## Abstract

Intermittent fasting (IF) is a popular intervention used to fight overweight/obesity. This condition is accompanied by hypothalamic inflammation, limiting the proper signaling of molecular pathways, with consequent dysregulation of food intake and energy homeostasis. This mini-review explored the therapeutic modulation potential of IF regarding the disruption of these molecular pathways. IF seems to modulate inflammatory pathways in the brain, which may also be correlated with the brain-microbiota axis, improving hypothalamic signaling of leptin and insulin, and inducing the autophagic pathway in hypothalamic neurons, contributing to weight loss in obesity. Evidence also suggests that when an IF protocol is performed without respecting the circadian cycle, it can lead to dysregulation in the expression of circadian cycle regulatory genes, with potential health damage. In conclusion, IF may have the potential to be an adjuvant treatment to improve the reestablishment of hypothalamic responses in obesity.

## Introduction

The neuronal circuits controlling food intake and the endocrine mechanisms involved in this complex modulation network have been widely investigated to clarify the factors associated with the regulation of energy homeostasis. The hypothalamus is considered the central point of this regulatory system. Therefore, impairment of the hypothalamic response generated by signaling disruption in crucial signaling molecules has been associated with the development of morbid obesity, highlighting the importance of controlling the hypothalamic function for health (1–3).

Different central nervous system regions mediate the regulation of food intake, body weight, and energy homeostasis. In this context, the mid-basal portion of the hypothalamus, where the arcuate nucleus is located, is composed of different subpopulations, including the orexigenic neurons, which are directly involved in the hunger stimulus, and also anorectic neurons, which are mainly involved in response to satiety signals (4). The agouti-related peptide (AgRP) orexigenic neuron and the pro-opium melanocortin anorectic neuron (POMC) are two essential components of energy expenditure, hunger, and satiety control neurocircuits, integrating central and peripheral energy status with metabolic signals (5). It is essential to highlight that the hypothalamus contains other neuronal groups involved in controlling food intake and energy expenditure (6), which are not the focus of this review.

Agouti-related peptide orexigenic neurons co-express the messenger ribonucleic acid (RNA) for the neuropeptide Y (NPY) and the neurotransmitter gamma-aminobutyric acid (GABA). Studies reveal that the intracerebroventricular administration of AgRP (7) or its overexpression is associated with increased food intake (8). In contrast, POMC anorectic neurons are co-located with those expressing the cocaine-and amphetamine-regulated transcript (CART) (2, 9). After its synthesis, POMC is cleaved by different enzymes, generating several peptides responsible for the POMC functions (10). Neurons expressing endogenous melanocortin ligands for POMC and AgRP neuropeptides (antagonists) and neurons containing melanocortin receptors compose the central melanocortin system (8, 11). This system is strictly involved in the control of food intake, glucose metabolism, and energy homeostasis (12, 13), in conjunction with anorectic hormones, primarily leptin and insulin, composing a complex neuroendocrine system to maintain the correct energy and body weight balance, as recently described by Yang et al. (14).

Both POMC and AgRP neurons have the leptin receptor (LepR). When leptin binds to POMC neuronal cell receptors, neuronal depolarization and activation initiate multiple signal translations related to satiety responses. The leptin-mediated signaling is transduced into the nucleus, producing the anorexic POMC and CART neurotransmitters (15, 16). In addition, a cross-inhibitory reaction between AGRP and POMC neurons induces a reduction in orexigenic neurotransmitters in the AGRP neurons.

Insulin is also a crucial hormone for maintaining energy homeostasis by inhibiting pathways associated with NPY/AgRP neurons and their ramifications (16, 17). Therefore, impairments in the central signaling pathways of insulin (18–20) and leptin (21, 22) are associated with energy imbalance and obesity development. In this context, intermittent fasting (IF) is a protocol popularly used as a strategy to promote weight loss (23) and has become a tremendous scientific topic of interest to elucidate the mechanisms that regulate the hypothalamic molecular responses that will reduce body weight and prevent obesity (24, 25).

Previous investigations in human and animal models analyzed the effects of IF on leptin and insulin sensitivity (26, 27), inflammatory pathways (28, 29), the brain-microbiota axis (30, 31), circadian cycle (32, 33), and autophagic pathway (34). All these factors seem to be related to adaptations in POMC and AgRP neuropeptides (35) that can improve energy homeostasis through pathways that are not yet fully understood. The present review explored the molecular and physiological adaptations of leptin, insulin, POMC, and AgRP neuropeptides to IF protocols, mostly performed in animal obesity models.

### Leptin and Insulin in Energy Homeostasis: Molecular Pathways Linked to Pro-opium Melanocortin Anorectic Neuron and Agouti-Related Peptide Responses

Insulin and leptin are the main anorectic hormones that act on the arcuate nucleus, activating POMC neurons and inhibiting AgRP neurons (36). Several studies indicate that the loss of hypothalamic insulin signaling (18–20) and leptin (21, 22) can induce changes in energy homeostasis, excessive food intake (hyperphagia), and body weight gain, leading to obesity development. The arcuate nucleus is densely rich in leptin receptors (37). The intracellular signaling cascade begins after leptin binds to its receptors in neuronal cells. An internal conformational alteration in the LepR attracts the next downstream protein, JAK2 (Janus kinase 2) (38). JAK is a cytoplasmic cytokine receptor that can autophosphorylate and promote the phosphorylation of its intracellular tyrosine residue Y-938, associated with the recruitment of the phosphatase SHP2 and its extracellular regulator ERK2, and of the residue Y-1077, which recruits the STAT5 transcriptional and signal transduction activator pathway. The primary effects of leptin on energy homeostasis involve the phosphorylation of the Y-1138 tyrosine residue, which creates a STAT3 binding and recruitment site (39). After its binding and subsequent activation, the STAT3 is transferred to the nucleus of the neuronal cell and promotes the transcription of genes, such as the neuropeptide POMC (40, 41).

Regarding insulin, despite having been discovered in 1921 (42), the complete elucidation of its molecular signaling is still in progress. However, it is known that the insulin receptor (IR) is a tetrameric enzyme that comprises two extracellular alpha subunits and two transmembrane beta subunits. Once the hormone interacts with its receptor, there is activation and consequent phosphorylation of the generated substrates (IR family), leading to activation of its main pathway, the phosphoinositide 3-kinase (Pi3K) pathway, a heterodimeric lipid kinase that binds to tyrosine residues *via* its SH2 domain, generating PI membrane phosphates with PkB/Akt recruitment (43).

Both insulin and leptin can stimulate the Pi3K pathway in the arcuate nucleus with subsequent phosphorylation of their target proteins, leading to hyperpolarization and activation of POMC neurons (44) and inhibition of AgRP (17). Mice with genetic Pi3K deletion in POMC cells did not show activation of POMC neurons in response to insulin or intracerebroventricular leptin administration (45). However, Pi3K deletion in AgRP neurons seems to induce energy expenditure reduction, insulin and leptin resistance, and weight gain (17).

The mechanistic target of rapamycin (mTOR) is one of the Pi3K target molecules through activation of Akt in the hypothalamus (Pi3K/Akt/mTOR pathway) (46). Both leptin and insulin activate hypothalamic mTOR (47, 48), a serine-threonine kinase with an essential role in brain development (49), which is found in approximately 90% of NPY/AgRP neurons and 45% of POMC/CART in the arcuate nucleus (50). mTOR is known for acting as a metabolic energy sensor and can integrate the variations in the nutrient serum levels with the endocrine responses (51). Thus, in food deprivation (fasting) and with drastic drops in serum glucose and insulin levels, there is a decrease in the phosphorylation of the mTOR active form. On the other hand, increased serum levels of leptin (51) and insulin (52) leads to increased mTOR protein content and expression and reduced food intake in the fed state.

The study of Kocalis et al. (53) observed that the deletion of Rictor-mTOR complex (mTORC2) activation, specifically in POMC neurons, can induce hyperphagia and increase adiposity. Interestingly, the specific deletion in AgRP neurons did not affect energy balance, although it led to mild glucose intolerance. It is known that the p70S6k-mTOR kinase further leads to phosphorylation of AMP-dependent protein kinase α2 (AMPK α2) on serine 491, inhibiting its action and thus limiting the effects of leptin on food intake (52). Thus, in parallel with mTOR activation by food intake, the anorectic hormones leptin and insulin reduce the AMP-dependent protein kinase (AMPK) activity, specifically the AMPKα2 subunit (54, 55). Like mTOR, AMPK is also known as a metabolic energy sensor, being considered an essential protein in the complex system of intracellular energy regulation, which is based on the adenosine triphosphate (ATP)/adenosine diphosphate (ADP) ratio (56).

In conditions of depletion of energy reserves such as hypoglycemia and fasting, AMPK is activated in the hypothalamus (57, 58), leading to increased gene expression of NPY/AgRP in neurons and stimulating food intake (54). AMPK is inhibited in the hypothalamic arcuate nucleus in the fed state in response to increased leptin, insulin, and high levels of serum glucose (54), which consequently inhibits the autophagic pathway in NPY/AgRP neurons, leading to a reduction in food intake by inducing the feeling of satiety and, thus contributing to the eutrophic phenotype (44, 50, 59). Other molecules and pathways are also stimulated by anorectic hormones and contribute to energy homeostasis. Further details about the molecules involved in the signaling pathway of insulin and leptin actions in POMC and AgRP neurons in eutrophic conditions were described in the review article by Varela and Horvath (16).

**FIGURE 1:**
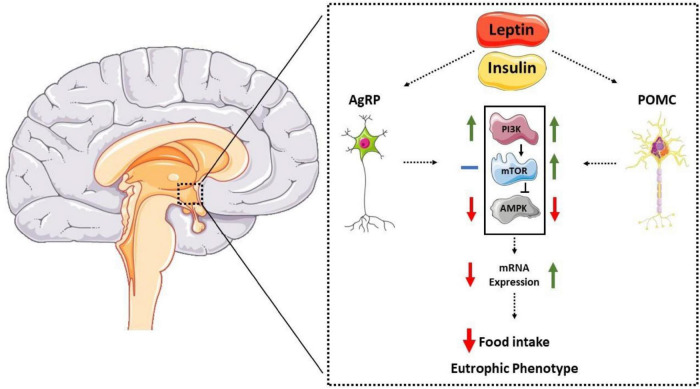
Representative diagram of the interaction between anorectic hormones, molecular pathways, and neuropeptides POMC and AgRP. Anorexigenic hormones (leptin and insulin) act on the hypothalamic arcuate nucleus, reducing AMPK activation in AgRP neurons, thus reducing its expression. In POMC neurons, hormones increase mTOR activity and reduce AMPK, increasing its expression and reducing food intake and eutrophic phenotype.

Regarding the PI3K-mTOR-AMPK pathway, anorexigenic hormones increase the activity of the Pi3K and mTOR pathways, leading to the activation of POMC neurons (44, 45). Additionally, leptin and insulin reduce AMPKα2 activity in the hypothalamic region (15, 54). The p70S6k-mTOR kinase can inhibit the AMPK pathway in the hypothalamus, acting as a counter-regulatory protein (52). The mechanisms by which these molecules modulate the expression of hypothalamic neuropeptides are not fully understood; however, evidence suggests that the autophagic pathway plays a crucial role in this regulation (59–61). Furthermore, Claret et al. (55) showed that mice with genetic deletion of AMPKα2 in AgRP neurons were grown with the eutrophic phenotype. Interestingly, the specific genetic deletion in POMC neurons led to increased body fat and reduced caloric expenditure despite remaining sensitive to leptin. These results suggest that AMPK also plays a regulatory role in POMC neurons by unknown mechanisms. Therefore, evidence suggests that the hypothalamic autophagic pathway is crucial for activating orexigenic and anorectic neurons (59, 61, 62). Figure 1 summarizes the data described so far.

### Autophagic Pathway in Pro-opium Melanocortin Anorectic Neuron and Agouti-Related Peptide Neurons: A Pivotal Point in Energy Homeostasis

It is well known that the neuronal autophagic pathway is crucial for maintaining cellular homeostasis both under basal conditions and in response to stress signals (63, 64). Several studies indicate that the imbalance between activation and inhibition of the autophagic pathway in the central nervous system (CNS) is associated with dysregulation of body energy homeostasis and obesity induction and a greater predisposition to the development of various neurodegenerative diseases (61, 65, 66).

Classically, autophagy can be divided into microautophagy, chaperone-mediated autophagy, and macroautophagy, the latter being the most prevalent and commonly referred to as autophagy (67, 68). The autophagic process begins with capturing cytoplasmic organelles or macromolecules surrounded by a vesicular membrane lining called the autophagosome, which fuses with the lysosome to form autophagolysosome (or autolysosome), and lysosomal enzymes then degrade the sequestered material (69). The autophagosome formation begins with a pre-phagophore structure, which elongates and expands to form the phagophore, which, in turn, will mature in the membrane vesicle, surrounding the substrate that will be degraded (69).

**FIGURE 2:**
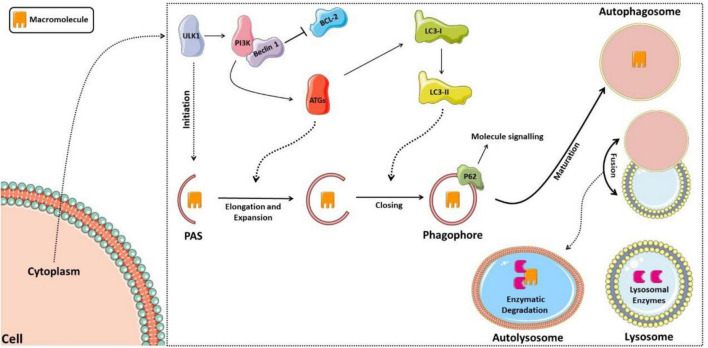
Schematic model of the autophagic pathway. The process starts with activation of the ULK1 complex, then activation of phosphatidylinositol 3-kinase (PI3K), which forms a complex with Beclin 1 after it dissociates from lymphoma B cell 2 (BCL-2). Thus, the complex formed activates several proteins of the autophagic family (ATGs), which participate in the elongation of the phagophore and activation of the LC3-I protein (light chain 3 of protein 1 associated with microtubules), forming LC3-II, responsible for closing the phagophore and interacting with the p62 protein, signaling the material to be degraded. The phagophore matures into the autophagosome, which fuses with the lysosome forming the autolysosome, in which lysosomal enzymes will then degrade the sequestered material.

The regulation of this entire autophagic process occurs by activating autophagic molecular complexes, starting with activation of the ULK1 (Unc-51 like autophagy activating kinase 1) complex, followed by the activation of phosphatidylinositol 3-kinase (PI3K), which forms a complex with Beclin 1 after dissociating from lymphoma B cell 2 (BCL-2) (70). Thus, the formed complex activates several proteins of the autophagic family (ATGs) that participate in phagophore elongation and activate the LC3-I protein (light chain 3 of protein 1 associated with microtubules), forming LC3-II. LC3-II is responsible for closing the phagophore and interacting with the p62 protein, which targets the material that the autolysosome will degrade (71, 72). Figure 2 shows the schematic model of the autophagic pathway.

Evidence suggests that the hypothalamic autophagic pathway is crucial in activating orexigenic and anorectic neurons (59, 61, 62). AMPK is an activating molecule of the autophagic pathway, while mTOR leads to inhibition (73). AMPK can activate the autophagic pathway *in vivo* and *in vitro* by phosphorylating raptor-mTOR (mTORC1) (74) and starting the autophagic pathway initiator complex ULK-1 in AgRP neurons (59). Therefore, AMPK and mTOR directly interact to regulate the autophagic pathway through its complex initiator, ULK-1 (73).

Specifically, in AgRP neurons, the deletion of Rictor (rapamycin-insensitive companion of TOR), a key molecule in the regulation of the MTORC2 complex, did not change the energy balance (53). However, the deletion of AMPK led to the eutrophic phenotype (55), suggesting that AMPK plays a more expressive role than mTOR in the energy regulation pathways of this neuronal subgroup. An elegant study published by Kaushik et al. (61) showed that inhibiting the autophagic pathway, specifically in AgRP neurons, in both cells and mice, through the Agt7 gene deletion, significantly reduces food intake and adiposity. In POMC neurons, studies with knockout mice showed that the activity of mTORC1 and mTORC2 complexes are vital factors for the anorectic effects induced by leptin on the neuron and maintenance of the eutrophic phenotype (53, 75).

While the reduction in AMPK activity in AgRP (59) neurons and the increase in mTOR activity in POMC neurons (53, 75) lead to the eutrophic phenotype and considering that AMPK inactivation and mTOR elevation lead to inhibition of the autophagic pathway (73, 76), hypothetically, inhibition of the autophagic pathway in neurons may be associated with the eutrophic phenotype. However, a study showed that the selective deletion of autophagy-related protein 7 (Atg7) in mouse POMC neurons, interestingly, leads to a reduction in melanocyte-stimulating hormone (MSH) and is also associated with increased adiposity and food intake through mechanisms involving resistance to lipolysis (60). In addition, Meng and Cai (77) observed that the suppression of Atg7 in the mediobasal hypothalamus using site-specific lentiviral delivery of shRNA, without distinction of neuronal subgroups, was accompanied by an increase in hypothalamic inflammation, with activation of IKKB and, consequently, increased food intake and reduced energy expenditure (77).

Supporting these data, the AMPK deletion specifically in POMC neurons (55) and the RICTOR/mTORC2 deletion in the arcuate nucleus (53) are also associated with reduced caloric expenditure and obesity. Together, these data reveal that AMPK and mTOR are correlated with the autophagy pathway, which orchestrates a series of coordinated molecular phosphorylations in neuronal subgroups to provide adequate control of hypothalamic inflammation and energy homeostasis, reinforcing that the hypothalamic molecular pathway of obese individuals needs to be further investigated. Table 1 presents the metabolic phenotypes found according to the deletion or inhibition of molecular pathways in the neuronal subgroups.

**TABLE 1:** Molecular pathway deletion or inhibition in the neuronal subgroups and the outcomes.

Neuronal target	Deletion or inhibition of neuronal molecular pathways	Species	Outcome	References
AgRP	AMPK	Mice AMPKα2KO	Eutrophic phenotype, light level of glucose intolerance	(55)
	mTOR	Mice lacking Rictor in AgRP	mTORC2 did not change energy homeostasis	(53)
	Autophagic pathway	Atg7F/F-AgRP-Cre mice	Better food intake control and eutrophic phenotype	(61)
POMC	AMPK	Mice AMPK α2KO	Hyperphagia, obesity, hyperglycemia	(55)
	mTOR	Mice lacking Rictor in POMC and C57BL/6JPOMC-rptor-KO	Rictor/mTORC2: decreased energy expenditure and induced obese phenotype, did not induce leptin resistance mTORC1: limited ROS capacity to inhibit food intake	(53, 75)
	Autophagic pathway	Atg7*^F/F^*-POMC-Cre mice	Limited lipolysis capacity and obese phenotype	(60)
NPY/AgRP and POMC simultaneously	AMPK	*In vitro* and *in vivo* (male C57BL/6)	Dysregulation of autophagic pathway and reduction in body weight	(59)
	mTOR	Mice lacking Rictor in all neurons	Increased adiposity, glucose intolerance, leptin resistance	(53)
	Autophagic pathway	Mediobasal hypothalamus Atg7 K_*D*_ mice	Hyperphagia, reduced energy expenditure, and hypothalamic inflammation	(77)

*AMP-dependent protein kinase (AMPK), Rapamycin target protein (mTOR), Reactive oxygen species (ROS).*

### What Is Intermittent Fasting?

Intermittent fasting (IF) and caloric restriction are two distinct forms of dietary restriction associated with improving several metabolic parameters, including body weight control (78). Previous studies have shown that the obligation to maintain a daily calorie restriction reduces adherence to the caloric restriction protocols (79). Thus, the presence of *ad libitum* feeding windows in IF protocols emerged as an alternative protocol for dietary restriction interventions. The stress promoted by the low caloric intake is replaced by the metabolic stress induced by intermittent windows of prolonged fasting or alternate days of deficient caloric intake (78, 80). However, it is essential to highlight that both interventions must be carried out with professional supervision. Overfeeding episodes can occur after the fasting window, with the risk of developing eating disorders such as binge eating (81).

Furthermore, caloric restriction programs can also increase the predisposition to the development of psychological disorders (79). The review of Cerqueira et al. (82) pointed out that in animals fed with standardized diets balanced in macro and micronutrients, calorie restriction protocols with daily consumption of 40–60% of energy requirements are associated with micronutrient deficiencies. Deficiency of vitamin B12 and vitamin K, among others, depending on the diet consumed, is observed when the restriction protocols are chronically applied without supplementation with vitamins and minerals (79).

Despite its high popularity, there is no standardization of IF protocols (80). It is established that protocols do not impose water restrictions. All include periods of food restriction, which may refer to total deprivation from food consumption during some hours of the day (fasting window) or a full day containing no-energy food. Recently, some papers (80, 83) have been considering IF in three specific categories: (a) Complete Alternate Day Fasting (ADF) – consists of days with *ad libitum* feeding intercalated by whole days of food restriction, (b) Modified alternate-day fasting (MADF) or alternate-day modified fasting (ADMF) – with two non-consecutive days of total food restriction within the week, or two days of food intake of about 20% of the total caloric necessity with meals distributed throughout the day (c) Time-restricted feeding (TRF) – consisting of a protocol with a fasting window (usually 16 h) followed by a food intake window of approximately 8 h, with the meals distributed within this period, according to individual needs. The main point of the protocols is not to change the average weekly caloric intake but to change the frequency of food consumption (78, 80).

In this sense, the application of IF protocols generally does not change the average calorie intake due to post-fasting compensatory overfeeding. Thus, only a slight reduction in the average percentage of daily intake (84, 85) contributes to the protocol being considered an alternative strategy to improve weight loss and induce positive metabolic adaptations generated by energy stress (84, 85). However, it is essential to mention the warning that IF protocols are not recommended in cases of malnutrition, pregnancy, gastric ulcers, elite athletes, and patients at risk of hypoglycemia, among others (86–88).

Unlike the globally disseminated IF protocols for weight loss and improvement in health-related aspects (23, 24, 89), Ramadan fasting is a protocol with spiritual purpose practiced by Muslim followers of Islam (90). Once a year, according to the Islamic calendar, Muslims abstain from any food or drink, including water, during the period of daylight, having all their meals in the evening or just before sunrise (91). This practice extends for about 30 consecutive days once a year during the Islamic lunar month, which can occur in different seasons depending on the year (92) and the latitude of the geographic region. The fasting window can vary from 11:00 am to 6:00 pm (93). During this practice, most Muslims eat about two bulky meals within 24 h, one just after sunset and the other just before sunrise (93), resulting in a slight but significant reduction in the total calorie intake (94, 95) regarding loss or maintenance of body weight (96). Table 2 illustrates the main differences between intermittent fasting, caloric restriction, and Ramadan fasting.

**TABLE 2:** Main differences between intermittent fasting, caloric restriction, and Ramadan fasting.

	Intermittent Fasting protocols	Caloric restriction	Ramadan fasting
Caloric intake	Considering the food consumption throughout the week, there is a slight caloric intake restriction (97)	TRF: intake of 40% to 60% of the total energy expenditure, or daily restriction of 500 kcal to 1000 kcal (82) ADF: includes days containing absolute fasting of food (83). MADF: includes 2 days a week with no-energy intake or days with severe restriction of food intake (less than 25% of daily necessity) (80)	Slight caloric intake restriction (300 kcal) (95)
Meals daily distribution	2 to 7 food restriction windows weekly. Usually composed by 16 h-fasting or 2 days of the week with a caloric intake lower than 20% of the TCI (78)	Daily caloric restriction with a variable number of meals (TRF, MADF) (82) or days without any meals (ADF, ADF)	Fasting during daylight period (from 11 AM to 6 PM). Generally two meals a day, one after sunset and one before sunrise (93)
Related risks	Binge eating and hypoglycemia (81)	Vitamin and mineral deficiency (82)	Risk of dehydration and accidents at work (98)
Liquid intake	No restriction (84)	No restriction (82)	Restricted, including water restriction. Liquid intake is allowed only at night (90)

### Intermittent Fasting as a Possible Adjuvant in the Treatment of Obesity: Modulations in Neuroinflammatory, and Leptin and Insulin Pathways

Obesity is a multifactorial disease usually associated with hyperphagia, hyperinsulinemia, and hyperleptinemia. The high levels of leptin and insulin in the cerebrospinal fluid of obese individuals indicate a chronic state of resistance to the actions of these hormones in the CNS (19, 99). It is essential to highlight the diet quality profile as a significant possible factor in the pathophysiology of obesity. Increased exposure to a high-fat diet (HFD) is associated with a reduction in hypothalamic mTORC1 and leptin resistance (100). There is evidence that an acute lipid infusion for 24 h or exposure to a HFD over 8 – 20 weeks induces markers of inflammation in the hypothalamic NPY/AgRP neurons, which may contribute to a significant alteration in NPY/AgRP expression or content (101) and also, 6 days of exposure to a high-fat diet can induce leptin resistance in mice with a predisposition to obesity (102).

Several studies indicate that the practice of IF for periods longer than 1 month can improve insulin resistance and reduce its serum levels, contributing to the regulation of glucose metabolism (26, 103–105). A recent meta-analysis evaluated 545 participants, most overweight or obese, and observed that IF protocols are associated with reducing the body mass index (BMI) and leptin serum levels, lowering fasting blood glucose, and improving insulin resistance. These results suggest that IF may contribute to prevention/improvement in the resistance of the anorectic hormone observed in obese individuals (106).

Although not fully elucidated, the mechanisms by which IF acts in the insulin signaling pathway are probably different from those observed in caloric restriction protocols since benefits associated with IF can be observed even when there is no reduction in calorie intake and weight loss (84, 103, 107, 108). In addition, there is some evidence that IF protocols may produce more significant beneficial effects on glucose regulation and fasting insulin (103, 108).

It is known that obesity is associated with the chronic low-grade inflammatory process, not only peripheral but also central, highlighted by increased expression of several inflammatory proteins related to impairments in the hypothalamic signaling of leptin and insulin, such as the suppressor of insulin signaling cytokine 3 (SOCS3) (103, 109, 110). Despite SOCS3 being part of a negative feedback system related to this signaling cascade, when it reaches a high concentration induced in an inflammatory scenario, SOCS3 significantly impairs the anorexic leptin cascade. This cytokine can bind to an intracellular region of LepR, attenuating the ability of JAK2 to autophosphorylate and recruit the STAT3 pathway (111). In addition, the C-terminal portion of SOCS can recruit the ubiquitin transferase system, promoting the degradation of JAK receptor complexes (112). Thus, SOCS3 impairs the reduction in the activity of the AMPK protein threonine 172 by leptin (113), stimulating autophagic activity in AgRP neurons and appetite (59). It is also known that SOCS3 can impair the insulin signaling pathway by binding directly to the insulin receptor (114) and/or degrading both substrates of insulin receptors 1 and 2 (IRS1/2) (115). The study of Mori et al. (109) observed that hypothalamic suppression of SOCS3 could prevent central insulin resistance generated by the chronic high-fat diet.

In this sense, although several studies show that IF protocols can reduce plasma levels of pro-inflammatory proteins in obese or overweight individuals (27, 116), few studies have assessed the adaptation of inflammatory proteins in the hypothalamic region. Spezani et al. (27) evaluated the effects of a 24-h fasting protocol interspersed with days of *ad libitum* high-fructose diet in mice with induced obesity (eight-week protocol with a high-fructose diet). After 4 weeks of intervention, a reduction in the expression of hypothalamic SOCS3 was observed. However, animals fed a standard diet and submitted to an IF protocol showed an increase in SOCS3 compared to control animals with a standard diet without applying the IF protocol (27). Controversially, the study of Zangh et al. (117) did not observe changes in the expression of hypothalamic SOCS3 or alteration in plasma insulin in female mice fed with a standard diet and submitted to chronic IF protocols for 24 h performed only one to two times a week, during a period of 13 or 42 days.

It is also known that the increase in tumor necrosis factor-alpha (TNFα) attenuates the anorectic effect of leptin and increases the expression of SOCS3 in the hypothalamus (118). Despite studies showing that caloric restriction (116) and Ramadan fasting (119) can reduce plasma TNFα levels, particularly in obese or overweight individuals, a recently published meta-analysis (28) evaluated serum levels of inflammatory markers in response to different IF or caloric restriction protocols. After applying the exclusion criteria, the meta-analysis included only one study with obese individuals and IF (alternating every 24-h between consuming 25% or 125% of energy needs), which did not reduce TNFα levels (120). However, it is crucial to consider the lack of papers published in this area.

Regarding animal studies, Spezani et al. (27) evaluated the effects of IF for 24 h. The authors observed that obese mice submitted to fasting curiously showed a greater expression of hypothalamic TNFα when compared to the control group. Therefore, the data are still contradictory, and further studies are needed to assess the content and expression of TNFα, specifically in the hypothalamus of obese animals submitted to different IF protocols.

Another relevant inflammatory pathway involved in the etiology of obesity is the IKKb/NF-kb pathway (110). Zhang et al. (110) showed that mice submitted to a high-fat diet developed obesity accompanied by increased concentrations of IKKB, which can activate the nuclear factor kb (Nf-kb), leading to endoplasmic reticulum stress in the hypothalamus and consequent resistance to leptin and insulin (110). IKKB phosphorylation in the mid-basal portion of the hypothalamus can impair the action of insulin by inducing tyrosine phosphorylation and the consequent inactivation of the insulin receptor (IR). In addition, it can limit the activity of its target proteins: phosphatidylinositol-3-kinase (Pi3K) and protein kinase B (Akt) (110, 121), which are involved in the control of the hypothalamic autophagic pathway (70). In a complementary way, the increase in IKKB contributes to the elevation of the expression of hypothalamic SOCS3 (110), impairing the central signaling of leptin and insulin (41, 122). However, although IKKB is a protein widely studied in obesity models, no investigations have evaluated IKKB in the hypothalamic region in response to IF protocols.

The IKKb/NF-kb inflammatory cascade can also be activated by lipopolysaccharides (LPS) when bound to their Toll 4 membrane receptor (TLR-4) (123, 124). Previous studies have shown that obese individuals present increased levels of LPS in the bloodstream, causing a condition called metabolic endotoxemia, which is associated with systemic inflammation and an increased risk of developing chronic diseases (125), favoring the development/worsening of obesity (125, 126). Additionally, prolonged treatment with LPS seems to increase JNK and limit the hypophagic effects in response to central insulin administration, regardless of the increase in body weight (127). Although not fully understood, the increase in LPS plasmatic levels is probably due to intestinal dysbiosis and changes in the permeability of the intestinal wall (128, 129).

Dietary factors seem to modulate endotoxemia, and the use of prebiotics could contribute to attenuating its progression (31), while chronic exposure to a high-fat diet intake could worsen progression (125). In this sense, a recent review article proposed that IF protocols can also be used as a nutritional strategy, affecting the brain-microbiota axis of obese individuals (31). An elegant study demonstrated that the removal of the intestinal microbiota with the use of antibiotics reduced the protective effects of IF on the cognitive function of the evaluated mice, with the subsequent administration of microbiota metabolites, such as short-chain fatty acids and 3-acid propionic indole, which were able to improve cognitive function and insulin sensitivity (30). Together, we hypothesized that IF might play a supporting role in attenuating inflammation in the CNS through actions on the microbiota-brain axis. However, this hypothesis needs to be evaluated.

Additionally, it is known that during acute fasting periods, there is an increase in β-hydroxybutyrate (βHB) production (130), leading to increased phosphorylation of IRS1 and Akt in their active forms, a reduction in serum insulin levels, and a better response to the intraperitoneal insulin tolerance test (131), as well as being able to modify hypothalamic leptin and insulin signaling pathways in type 2 diabetic rats (132). It is essential to highlight that βHB is also involved in inflammatory control (133). The oral administration in Crohn’s disease patients exerts an anti-inflammatory response through downregulation of NF-kb (134). Cerniuc et al. (135), evaluating an IF protocol (2 non-consecutive days of total fasting per week) in healthy women, also identified a significant increase in blood βHB levels. To date, despite not directly evaluating insulin response associated with βHB production and hypothalamic responses in IF protocols, data suggest that the increase in butyrate levels may also contribute to improving insulin sensitivity in response to IF.

Although the relationship of neuroinflammation with insulin and leptin signaling in response to IF protocols need to be further explored, data suggest that: (1) the mechanisms of action of IF seem to be different from those observed in calorie restriction protocols (103, 108); (2) IF appears to be able to improve insulin and leptin sensitivity (105, 106); (3) IF seems to be able to modulate inflammatory pathways in the brain (27, 29) and attenuate the levels of LPSs in the plasma (29), which we hypothesize could be associated with an improvement in hormonal and neuronal sensitivity; and (4) the increase in βHB production in IF (135) may also contribute to better insulin sensitivity considering the relationship of βHB and the insulin pathway (131, 132).

### The Influence of the Circadian Cycle on the Modulation of Leptin and Insulin Pathways in Different Intermittent Fasting Protocols

Although several studies show that IF protocols are capable of improving insulin and leptin sensitivity, it is essential to emphasize that the time when the fasting window and the eating window are performed significantly interferes with metabolic responses and autophagic stimulation due to their influence on the hormonal rhythm guided by the circadian cycle (33, 136). However, the habit of skipping breakfast is associated with greater consumption of food at night (breakfast skipping and late-night eating pattern), increasing the risk of developing insulin resistance and cardiometabolic risk (33, 137, 138).

Jamshed and coworkers (33) submitted overweight or obese individuals to IF protocols. The authors evaluated the differences between the protocol carried out with the food window from 8 AM to 2 PM (Early Time-Restricted Food – eTRE) with a second protocol containing the last meal at 8 PM, both on a controlled diet. After four consecutive days of intervention, the authors observed increased BMAL1 expression in the morning, activation of Akt2, reduced fasting plasma insulin, and glucose concentrations in the eTRE group compared to the group that had the last meal at 8 PM (33). These results corroborate another study carried out with humans by the same research group that observed that the eTRE group improved insulin sensitivity, assessed by the glucose tolerance test, compared to the group fed at night (107).

The IF protocol performed without respecting the circadian cycle can induce a dysregulation in the expression of the circadian cycle leading to a significant increase in the natural peak of mRNA expression of genes involved in glucose regulation (i.e., *Gck, Slc2a2*, and *Pdk4*) and also lead to a higher plasma leptin levels when compared to an IF protocol applied to respect the circadian cycle (with a distributed feeding window in the active period of mice) (32). On the order hand, an IF protocol respecting the circadian cycle seems to reverse the obese and hyperphagic phenotype of heterozygous knockout mice of brain-derived neurotrophic factor (BDNF) and re-established insulin sensitivity and brain BDNF levels after 3 weeks of intervention (139). Thus, due to its influence on the circadian cycle, the effects of IF on endocrine responses and body weight may vary according to the time of day in which each food and fasting window is held. It seems better for healthy improvements not to skip breakfast and start the fasting window close to sunset to improve sensitivity to anorectic hormones and help prevent obesity (137, 140, 141).

Therefore, it is essential to point out that these data warn us regarding popular IF models disseminated in social media that encourage avoiding breakfast and starting the eating window at lunch. It is essential to reinforce the importance of a scientific basis to achieve better dietary prescriptions at the individual and population levels. This topic was deeply explored in the recent review published by Moon et al. (142).

### Intermittent Fasting and the Autophagic Pathway

Autophagy is an essential mediator of physiological responses associated with the generation of ROS and cellular protein damage (143), being directly involved in maintaining energy homeostasis through the increased expression of neuropeptides (144) and in the control of the neuronal inflammatory response (77). Energy stress and IF-induced oxidative stress can activate the autophagic pathway (145) through the increase in sirtuins (SIRTs) (146, 147), associated with increased phosphorylation of AMPK in threonine (148, 149), correlated with phosphorylation of the ULK1 protein and autophagic pathway (150). Therefore, the application of IF protocols is a non-pharmacological alternative capable of activating the autophagic pathway (33, 34).

During prolonged fasting, lower glycemic values and changes in the adenosine monophosphate/adenosine triphosphate (AMP/ATP) ratio induce SIRTs activation in tissues such as the kidney, skeletal muscle, and blood samples from overweight individuals (145, 147, 151). Sirtuins are known to induce the autophagic pathway through phosphorylation of AMPK, FOXO1, or deacetylation of autophagic family proteins (145, 149). A study evaluating the IF and autophagic pathway in adults observed an increase in serum levels of SIRT1 and the autophagosomal membrane component LC3A, thus suggesting autophagic stimulation, accompanied by improved insulin sensitivity (33).

AMP-dependent protein kinase is an essential protein associated with the neuronal autophagic pathway and energy homeostasis (59). An interesting study by Kaushik et al. (61) observed that acute fasting could increase the content of free fatty acids in the hypothalamus, with consequent phosphorylation and activation of AMPK and ULK1, increasing autophagic flow in AgRP neurons with hunger induction. Furthermore, the authors observed that impairment of the autophagic pathway in cultures of hypothalamic cells through the deletion of the protein related to autophagy 7 led to a reduction in AgRP levels, food intake, and adiposity (61). The increased availability of fatty acids can induce the hypothalamic autophagic pathway and increase NPY expression (144).

Additionally, the acute fasting protocol can also lead to phosphorylation of mTOR and its target protein, ribosomal protein kinase S6 (S6K) at serines 240 and 244 in the hypothalamus, thus inactivating the mTOR/S6k pathway (50). During fasting, the increase in AMPK associated with the reduction in mTOR contributes to the regulation of food intake (50, 52) and activation of ULK1 and the autophagic pathway (150). These data corroborate the work of Chaix et al. (152), who evaluated IF protocols in obese mice, and observed an increase in the levels of the homolog of ATG8, Gabarap1, a key regulator of autophagic flow during fasting. The animals showed significant weight loss compared to the control group, although food intake did not show any significant difference.

Although several studies have shown the effects of caloric restriction programs or fasting periods on the autophagic pathway, it is essential to emphasize that, to our knowledge, few studies have assessed the impact of IF protocols on the autophagic pathway. To date, we understand that fasting periods in general lead to increased availability of free fatty acids in the hypothalamic region, reduced levels of glucose and serum amino acids, leading to activation of sirtuins (33) and the AMPK pathway (148), and inactivation of the mTOR pathway, thus stimulating the autophagic complex (50, 73). There are no studies evaluating the autophagic pathway in the hypothalamic nucleus in response to chronic IF protocols.

### Intermittent Fasting, Pro-opium Melanocortin Anorectic Neuron, and Agouti-Related Peptide Neuropeptides

The mechanisms by which IF alters the expression of hypothalamic neuropeptides are not fully understood; however, it is known that IF can improve sensitivity to leptin and insulin (106) and stimulate the autophagic pathway (33, 145), which is related to the activation of hypothalamic neuropeptides and energy homeostasis (27, 55, 59, 60). Another relevant factor is the increase in reactive oxygen species induced by the IF protocol (146). ROS in the hypothalamus is also a factor that leads to the electrical activation of neuropeptides POMC and inactivation of Npy/AgRP (153).

The suppression of reactive oxygen species decreases the activation of POMC cells and increases the activity of NPY/AgRP neuropeptides (153). During fasting, the mechanisms of oxidative protection performed in the mitochondria protect the exacerbated increase in ROS in AgRP neurons. Uncoupling protein 2 (UCP2) is a protein abundantly expressed in arcuate nucleus neurons associated with energy homeostasis and involved in controlling oxidative stress in mitochondria (154, 155). A study shows that the function of UCP2 *via* AMPK seems to be a key point for the electrical activation of NPY/AgRP neurons during a fasting period (156). In contrast, ROS levels are low during fasting in POMC neurons, and the transient increase in ROS favors satiety and the action of leptin *via* mTOR (75, 157). However, these data were evaluated using acute fasting protocols. There is still no evidence about the content of ROS in neuronal groups and its impact on energy homeostasis in response to IF protocols.

Low serum leptin values during fasting contribute to an increase in the expression of AgRP and NPY (156, 158, 159), stimulating the autophagic pathway and consequently inhibiting activation of the hypothalamic-pituitary-thyroid axis to reduce caloric expenditure and save energy (160). However, contrary to the results observed after a single fasting period window, the study of Chausse et al. (161) submitted eutrophic rats to a 24-h IF protocol and observed that rats that underwent IF for 3 weeks, even with an excellent response to leptin, curiously showed increased expression of the orexigenic neuropeptide AgRP both during fasting periods and on feeding days. Increased energy expenditure, reduced energy efficiency factor, and lower weight gain were also observed when compared to the control group. These findings suggest that AgRP neuron responses to the IF protocol may differ from those observed after a single fasting window, and further studies are needed.

It seems that during the IF protocol, the response of neuropeptides can change. A study with eutrophic mice evaluating the response to IF protocols after 13 days and again after 42 days found that the first intervention increased food intake in the feeding windows accompanied by an increase in NPY mRNA expression. However, interestingly, after 42 days, there was a reduction in NPY mRNA expression that returned to baseline values. There was no difference compared to the control group (without IF intervention), thus suggesting a new late hypothalamic adaptation of NPY in response to the chronic application of the IF protocol (117), which may be related to the popularly described hunger adaptation. Regarding POMC neuropeptides, there were no significant changes.

Additionally, the time that the fasting window is performed can also influence the expression of neuropeptides. Animals exposed to a high-fat diet for 2 weeks and then submitted to the IF protocol of 16 h for 1 week, with the feeding period performed during the rest period, disregarding the circadian cycle, showed increased hypothalamic expression of the orexigenic genes NPY and AgRP. Higher food intake and higher serum levels of leptin were also observed, thus suggesting possible resistance to leptin compared to animals that followed the IF protocol with the feeding window performed in the active period (referring to the night period for mice). Therefore, it can be concluded that when the IF is carried out with the food period during the rest period, disregarding the physiological circadian cycle, there is a possibility that the protocol can trigger dysregulation of the neuroendocrine mechanisms of hunger control, which may harm health (32).

Few studies have assessed the effects of IF and neuropeptide expression in obese individuals. Gotthardt et al. (35) studied obese mice submitted to an IF protocol where the mice were food deprived every other 24-h period beginning at 9:00 AM (fasting day), 2 h into the light cycle, for 4 weeks. The results showed increased expression of mRNA of hypothalamic NPY and increased energy expenditure compared to the control group that consumed a high-fat diet *ad libitum*. Regarding the expression of POMC neurons, the group that performed the IF showed a significant reduction in the expression of the POMC neuropeptide when compared to the control group with an *ad libitum* high-fat diet, which was also accompanied by a decrease in serum levels of leptin, improvement in insulin sensitivity, and weight loss.

Interestingly, a study evaluating the effects of IF in animals fed with a standard diet observed that after 4 weeks of application of the IF protocol (24 h fed, 24 h fasting), the group with standard diet and fasting presented reduced expression of POMC when compared to its respective control (standard diet without fasting) (27). However, the authors also looked at the effects of IF on two other types of diet: obese animals fed a high-fat diet and obese animals fed a high fructose diet. After 4 weeks of applying the IF protocol (24 h fed, 24 h fasting), the fasted obese animals showed increased expression of POMC in both protocols (27).

**FIGURE 3:**
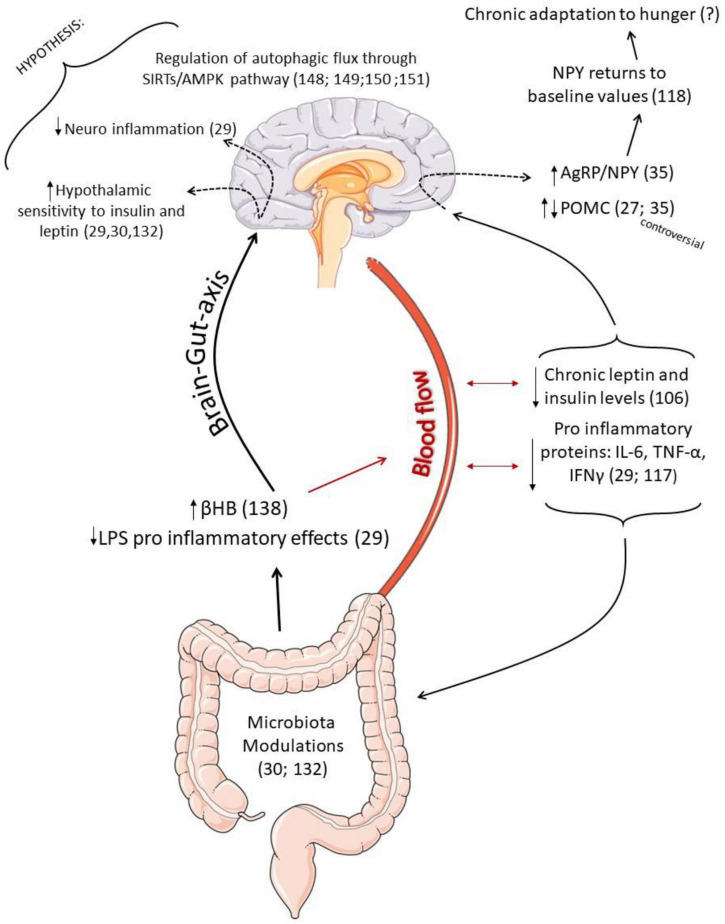
Schematic representation of the possible effects of intermittent fasting (IF) as an adjuvant treatment to partially rescue hypothalamic responses in obesity. Once IF prevents the pro-inflammatory effects in the hippocampus caused by LPS (29), and it is also able to increase the production of β-Hydroxybutyrate (βHB) (136), correlated to inflammatory control (134) and insulin sensitivity, it is possible to hypothesize that modulations in the microbiota may be helpful to reduce hypothalamic inflammation and increase hormonal sensitivity. Low serum leptin and insulin values during fasting contribute to an increase in the expression of AgRP and NPY. However, interestingly, the NPY mRNA expression returned to baseline values as a chronic response to IF, which may be is an adaptation to hunger. Furthermore, some studies found an increase in the expression of POMC neuropeptide, which is controversial. Such neuronal sensitivity to the IF protocol is perhaps also associated with better regulation of hypothalamic autophagic response since IF can activate the autophagic pathway in other tissues (146), and autophagic flux is an essential mediator of neuropeptide responses.

Therefore, we suggest that the content of neuropeptides in IF seems to occur differently from that observed in caloric restriction protocols, and the adaptations of the CNS seem to differ according to (1) the duration of time that the IF protocol is being applied, with the orexigenic neuropeptide NPY being able to return to baseline values as a late adaptation (117); (2) it is essential that the distribution of feeding and fasting periods respects the circadian cycle to avoid possible health risks (32); (3) concerning POMC neuropeptides, the IF protocol interestingly seems to reduce the expression of POMC neurons in some models (27, 35), but the results are still contradictory. Therefore, further studies are necessary to elucidate the effects of IF on hypothalamic responses and energy homeostasis. In addition, it is necessary to investigate long-term changes. Figure 3 summarizes the possible effects of IF as an adjuvant treatment to partially rescue hypothalamic responses in obesity.

## Conclusion and Future Perspectives

Evidence indicates that IF protocols can be used as a strategy to promote weight loss, as they induce an increase in energy expenditure (35, 161) and improve the peripheral response to anorectic hormones (33, 162), which can significantly interfere with the hypothalamic autophagic pathway (33) and also in the expression of neuropeptides (27, 35). Thus, the literature reviewed allows us to hypothesize that IF could help reestablish, at least in part, the control of hypothalamic molecular responses in obese individuals, alleviating neuroinflammation and improving hypothalamic sensitivity anorectic hormones, thus helping to enhance reestablishment of energy homeostasis. However, when the IF protocol is performed without considering the circadian cycle, it can impair energy metabolism regulation (32). These associations require more research, mainly when obese individuals submitted to long periods of IF are evaluated regarding the responses of the autophagic pathway and hypothalamic neuropeptides. In conclusion, considering the favorable results of IF in obesity, the protocol may be an adjuvant treatment to partially rescue hypothalamic responses in obesity.

## Author Contributions

All authors listed have made a substantial, direct, and intellectual contribution to the work, and approved it for publication.

## Conflict of Interest

The authors declare that the research was conducted in the absence of any commercial or financial relationships that could be construed as a potential conflict of interest.

## Publisher’s Note

All claims expressed in this article are solely those of the authors and do not necessarily represent those of their affiliated organizations, or those of the publisher, the editors and the reviewers. Any product that may be evaluated in this article, or claim that may be made by its manufacturer, is not guaranteed or endorsed by the publisher.
